# On classifying the field of medical ethics

**DOI:** 10.1186/s12910-017-0193-x

**Published:** 2017-04-27

**Authors:** Kristine Bærøe, Jonathan Ives, Martine de Vries, Jan Schildmann

**Affiliations:** 1 Department of Global Public Health and Primary Care, University of Bergen, Bergen, Norway; 2 Biomedical Ethics and Law, Centre for Ethics in Medicine, The University of Bristol, Bristol, UK; 3 Biomedical Ethics and Law, Department of Medical Ethics and Health Law, Leiden University Medical Center, Leiden, The Netherlands; 4 Professur für Medizinethik an der Wilhelm Löhe Hochschule, Fürth; Medizinische Klinik und Poliklinik III, Klinikum der Universität München, Campus Großhadern, Munich, Germany

## Abstract

In 2014, the editorial board of *BMC Medical Ethics* came together to devise sections for the journal that would (a) give structure to the journal (b) help ensure that authors’ research is matched to the most appropriate editors and (c) help readers to find the research most relevant to them. The editorial board decided to take a practical approach to devising sections that dealt with the challenges of content management. After that, we started thinking more theoretically about how one could go about classifying the field of medical ethics. This editorial elaborates and reflects on the practical approach that we took at the journal, then considers an alternative theoretically derived approach, and reflects on the possibilities, challenges and value of classifying the field more broadly.

## Introduction

In spring 2014, the Editorial Board of *BMC Medical Ethics* were invited to discuss a reorganisation of the journal into sections. The journal aims to publish “original peer-reviewed research articles in relation to the ethical aspects of biomedical research and clinical practice, including professional choices and conduct, medical technologies, healthcare systems and health policies”. The goal of the discussion was to devise sections that would allow submission of as wide a range of relevant articles as possible, but allowed submissions to be classified in a way that was useful for editorial purposes and comprehensible for authors and the readership. This collective deliberation resulted in a classification of four sections – each of which had specialist section editors attached:

- Ethics in biomedical research
- Ethics in clinical practice
- Ethics in public health, medical law, and health policy
- Methodology in bioethics

The approach taken was to divide the journal by areas of research, resulting in broad sections covering both theoretical and empirical approaches, such that an article submitted to a particular section would go directly to the relevant section editor after initial processing. The process and discussion was flexible, and the resulting classification was essentially a very practical solution to the challenges of managing the journal’s editorial process and content, with no intention of saying anything about the field in general. Given this, the sections used in the journal are open to revision, and will be revised as and when a better way to manage content is identified. In addition, authors who do not feel that one of the existing categories matches the content of their article can submit an article whilst leaving the decision about the section open to the editors.

While the above classification is strictly limited to the journal, the discussion about it also prompted a great deal of thought about how a more substantive, theoretically driven classification of the field might be undertaken, and demonstrated, for us, that there is no obvious, commonly shared perspective on how to coherently and adequately classify the various kinds of academic work that come under the very broad label ‘medical ethics’.

As we began to think more theoretically, it became clear that whilst the model developed for *BMC Medical Ethics* was the most appropriate and practical for the journal, this model could certainly be challenged. Recognising this, some of the journal editors were invited to write this editorial, which reflects on the possibilities, challenges, and value of classifying ‘medical ethics’. This editorial, then, reflects on the practical approach that we took at the journal and an alternative, theoretically derived approach which might also be used for other purposes (e.g., education, funding).

### Reflections on the practical approach for *BMC Medical Ethics*

By inviting the Editorial Board to participate in a discussion on a suggested set of sections, listening to their suggestions, and refining the ideas through discussion, the resulting classification can be described as the result of a deliberative and consensus-based approach. The value of such an approach can be debated – and one of its weaknesses stems from the putative arbitrariness that can be introduced by participants. The consensus reached depends on who takes part, and the very process that leads to a comprehensible and accepted consensus is also a process that could lead to over simplification and reduction to the lowest common denominator, resulting at worst in a failure to adequately cover the field, or perhaps at best a compromise that might be *acceptable* to many, but *pleases* few. A consensus approach can also serve to simply reinforce existing norms, as it will tend towards conservatism. However, the strengths of using a practical, consensus-based approach to classification, in this particular setting, are several, and outweigh these concerns. First, those involved in a well-designed, deliberative process are likely to accept the resulting classification as legitimate. This is a good way to establish ‘ownership’ with respect to the classification among the editors. Moreover, the process of consensus finding tends to result in a consensus that is comprehensible, and so there would be no need for further simplified description of sections in a way that would be more easily understood by authors and readers. Finally, as the editors in this particular setting can exercise judgment and flexibility in where to direct the articles, the problem of potential inadequacy in overall description of the field does not present any problem in terms of what the journal ends up publishing. Additionally, if the sections become, with time, unfit for purpose, they can be changed. With regard to the process undertaken by the journal, then, considering its purpose, these theoretically justified worries about the deliberative consensus based approach are mitigated.

In this editorial we want to draw attention to the broader challenges of classifying the field of medical ethics. We do so by exploring what a theoretical approach to classifying medical ethics could look like. This approach can be described as ‘theoretical’ because we derive conditions for classification from theoretical considerations about the nature of the field. We will provide pro- and con-arguments for this approach later in the text. For now, we start by laying out some general premises for the theoretically derived approach.

To classify the field of medical ethics for a practical purpose (i.e., with view to what use the classification will be put) is an example of ‘purposive classification’, where items are grouped according to features that will help achieve a specified aim. Given that our aim here is to classify the field of medical ethics in way that will enable it to be split up into discrete sections that will be useful to journal editors, authors, funders, students, and academic institutions, we need to take a very broad and inclusive approach. In *academic ethics,* the obvious purposive objects of classification are either activities (i.e., ways of ‘doing’ ethics) or concerns (i.e., topics of ethical interest) which are themselves interpreted, identified and expressed *conceptually*.

The inherent arbitrariness of a purposive classification can be explained in general by i) the fact that different purposes will lead to a different classification and, ii) even if the purpose is fixed, different justifications can be provided for what kind of features are most relevant to that purpose. We wish to stress that we acknowledge the implicit, constructed (non-natural) dimension of our approach, following from the fact that it is purposive; classification of medical ethics can always be presented by different sets of divided categories (i.e., in this context, sections of a journal). The conclusion we draw from this is that in order to *justify* one particular way of classifying a field of ethics, we must expose the conditions and assumptions involved in doing so. This means *we have to reflect carefully on the criteria that are relevant to a classification, as well as clarify particular points of view arrived at when trade-offs have to be made.* In doing so, we will be able to justify and specify requirements that, in turn, will determine the substantive classification at which we will ultimately arrive.

We also recognise that classifying a field of ethics brings with it the threat of potentially adverse political and ethical impact (which might indeed be even more threatening if the classification is mistakenly perceived as an articulated, natural classification). First, classification can be used - deliberately or not - as a tool of power. Those with the power to classify (for example to devise sections in a journal of medical ethics or to dictate thematic courses provided in bioethics education) also have the means to shape both research and education (directly or indirectly) through their decisions. If this is done in a way that merely promotes powerful individuals’ personal, narrowly defined academic interests, it can even amount to power *abuse*. The open invitation and flexible approach of *BMC Medical Ethics* to find a place for articles that are difficult to categorise is an exemplary way to mitigate precisely this kind of threat.

Second, another consequence of classifying the field of ethics is the risk that issues of ethical relevance fall outside, or in between, the defining categories. Such issues might end up going unpublished (or ignored) if they do not fit within the sections of the journal or the sub-disciplines that are taught in educational institutions. Consequently, there should be an ethical imperative incorporated into the business of classifying a field of ethics; *the classification must be adequate and sufficiently inclusive with respect to ethical concerns that already exist, and not hinder identification of new issues that might arise*. Again, *BMC Medical Ethics* provides an example of one way this challenge can be met. By inviting all kinds of submissions related to the field, and by being flexible in finding a home for them within the established sections, inclusivity is promoted.

Third, there is potential for unintended consequences in the real world. As an example, one should be aware of the potential ethical consequences stemming from an attempt to structure the field of ethics through a classification that, for example, focuses on theoretical approaches. Such a classification would promote a theoretical approach to doing ethics that may not accurately reflect the ethical challenges as they present themselves within particular contextualized practices (see, for example, Davies et al. [1]). Similarly, a classification that focuses on practical contextualised ethics runs the risk of denigrating more theoretical/abstract ethical reflection. Both these kinds of approach have import and value, and any kind of classification that leads to one being seen as less valuable than the other risks committing a wrong insofar as it begins to delegitimise other practices for no good reason. As a consequence, the academic goods that might flow from a having a variety of research practices might be lost, alongside the potential loss of impactful ethics research. Certainly, such unfortunate consequences cannot be accurately predicted, and are far from certain, but it may nonetheless be a good idea to be mindful of unintended consequences.

To summarise, in order to be perceived as justified, a classification strategy must expose the purpose of the classification, identify and make explicit the premises and assumption behind the classification, and explore trade-offs and explicit viewpoints taken towards these premises. As we see it, any approach to classifying the field of medical ethics for some strategic purpose should also be accompanied with self-reflective awareness of the three inherent threats described above: *power abuse, inadequate coverage of the field, and negative unintended impact on practice*. From this, substantive constraints on how to shape and determine a classification are derived. In the following, we will attempt to demonstrate how to do exactly that. We start by declaring the purpose of our classification, followed by laying out our initial reflections on a variety of premises for a purposive classification of medical ethics before deriving sections that are intended to adequately cover the whole field of medical ethics. We will then return to consider the threats described above and assess our suggested classification of medical ethics accordingly. Finally, we discuss the pros and cons of this theoretical approach compared to the practical deliberative approach used by the journal.

## The purpose of our theoretical classification of medical ethics

The importance of being clear about the purpose and process of a classification, and the challenges therein, is underscored by a recent attempt to develop a typology of bioethics by the American ‘Association of Bioethics Programme Directors’, as outlined in a recent paper in the Hastings Centre Report [2]. The authors’ purpose was to develop a typology that would assist in measuring success in Bioethics. As such, the typology proposed was based on differentiating the aims of different kinds of bioethics scholarship and the associated methods. Critical voices were published alongside the article, which challenged both the process, the scope, and the conceptual starting point and the purpose/value of the classification activity [3–6].

The *general* purpose of our theoretical classification is to conceptually divide the field of medical ethics into distinct sections that will be of use to stakeholders, which may include different journals, authors, readers, funders, educators and students. As such, our purpose is fundamentally different from that of the internal exercise for *BMC Medical Ethics*, which was focused on editorial, author and reader interests specific to that journal.

In this reflection we explore how we might develop a classification of the field that is unambiguously articulated and relevant and useful to journals, educators and funders. Bearing in mind the critical voices directed towards the paper by Matthews et al. [2], the challenge is to avoid categories that are too narrowly defined, and to ensure that the final classification encompasses everything that could reasonably be defined as medical ethics.

## ‘Scientific’ versus ‘Non-Scientific’ medical ethics

Since our purpose is to classify work on medical ethics in a way that can be used by, *inter alia,* journals and funders, the structures of classification must be compatible with a distinction between what counts as ‘research’ and what does not in the field of medical ethics. This will inevitably put some constraints on how we define the field, making our purposive classification perhaps more useful to journals, research funders and research led educators. Clearly, medical ethics does not become a research activity simply by being discussed by someone who is described as a ‘researcher’. More fundamentally, there must be a distinction between *bona fide* research and the mere expression of an opinion on an ethical matter.

We do not aim to contribute to on-going philosophical debates about the scientific nature of morality and ethics. Our aim here is much more modest; we will only point out a few uncontroversial characteristics of what would reasonably qualify as research in the field of medical ethics, and we make no claim that the list is exhaustive or authoritative. We state that research in medical ethics, understood as work that aims to contribute something new to the canon of knowledge, should be subject to a minimum of elementary criteria that apply to any kind of written work claiming to be’research’. If empirical data are involved, the study-design should be valid, the study should be (in principle) replicable to allow for critical scrutiny, and the conclusions should logically follow from the premises (for example, see Mertz et al. [7]). If the research is primarily theoretical, crucial assumptions should be clarified and claims should be consistently justified with arguments. If the work does not comply with these formal criteria for research, there are reasons to question the value of what is presented and the extent to which it can make a valuable contribution to our knowledge. As a very general claim, we take it that our general classification of the field should be formulated so as to cover any work on ethics enjoying a ‘research status’ according to these listed criteria.

From this general claim, a more substantial constraint on the classification of the field follows. In so far as medical ethics is understood as research, the field will also have to include self-reflexive, theoretical work on how to establish knowledge in ethics (i.e., how to determine the validity of ethical work), the relation between theory and practice when it comes to establishing justified knowledge, logical structures of justifying theories versus practical conclusions, conceptual analysis, and strategies for reaching a well justified classification of a particular field of ethics (like we attempt in this editorial) etc. Consequently, a classification of the whole field of medical ethics research needs to include sections that encompass philosophical, sociological and meta-ethical work on the conditions for medical ethics as a research activity itself.

## Ethics of practical approaches in ethics research

The previous paragraph on basic criteria for research relates to a fundamental question about the nature of medical ethics and paths of justification. While philosophical ethics is subject to theoretical constraints shaping the training of philosophers, the academic field of medical ethics is multi-disciplinary and includes researchers with training in various disciplines and methods. Does this fact detach this particular field of ethics from the logics that structure the justification of philosophical approaches?

First of all, there is not extensive agreement on how philosophy itself should justify its claims about ethics, neither substantially nor structurally. Let us concentrate, however, on one crucial question concerning justification within the field of medical ethics: does the fact that philosophy is not an unquestioned, authoritative discipline in medical ethics mean that academic medical ethics can abandon the requirement for theoretical normative justification, articulated through philosophical argument? If so, then our classification must admit a whole range of research activities on topics of medical ethical interest that do not engage directly in normative argument. If not, then we ought to include in our classification only those research activities that do result in a clearly expressed philosophical normative argument.

We hold that whilst reflective, theoretical work involved in articulating what goes into the ‘ought’ must have an essential and authoritative role in the structure of normative justification, research that does not directly engage with normative justification can nonetheless be classified as academic medical ethics so long as it is engaged with exploring and elucidating ethical norms, assumptions or lived moral experience.

Our argument for this is simple. Modern political and ethical theories rely on the assumption that individuals are moral equals. People can be confirmed as moral equals with respect to certain characteristics according to different normative theories. However, ultimately, it is the individual’s real world experiences of being treated as moral subjects and moral equals, despite their different characteristics, that constitutes the litmus test of how sensitive to ethics a society is. Academic work in support of developing normative solutions to complex ethical dilemmas cannot - from an ethical point of view - consistently promote solutions that do not consider or address the context of the problem or the impact of the proposed solution [8–10]. Therefore, in order for academic work on *practical* medical ethics to be ethical itself, it should recognise and respect the fact that individuals have real world lived moral experiences that ought to inform our thinking.

Our recognising the values of explicitly considering people’s experiences, and the value of theoretical work, has two important implications for our task of classifying the field of medical ethics. First, it stresses the importance of doing empirical research on people’s actual experiences in order to find acceptable ethical solutions. So, in our classification there must be room for contributions, for example, from social science in revealing people’s experiences and opinions on medical ethical issues. Second, a crucial task for theoretical approaches should be trying to understand why people do not feel like moral subjects and moral equals under certain regulations, to assess the relevancy of these reported experiences (as not everyone claiming to be treated unethically has reasonable grounds for claiming so) to raise concerns about practices that are accepted and taken for granted, and to give voice to those who cannot raise concerns themselves.

However, in order to preserve medical ethics research as a normative enterprise, we require non-arbitrary, context-independent normative theories that can be applied to concrete settings and be used to help critically analyse them. Ethical analyses do not emerge in a vacuum of interpretative or theoretical understanding, and ethical theories are needed to help us interpret and understand ethical experience. Indeed, our sensitivity for considering and characterising a situation as *ethical* in the first place - as well as our capacity to find practical solutions to these situation - can (at least partly) be explained according to a familiarity with ethical theories and general ethical norms. Therefore, our classification must also accommodate medical ethics research that deals exclusively with the development of ethical theory, or analyses that are context-independent.

## Theory versus practice

It follows from the previous paragraphs that as a subject for research, academic medical ethics should be considered to encompass production of both empirical and theoretical knowledge, but always with a view to informing normative argument. We argued above that theoretical (or philosophical) approaches are authoritative compared to empirical findings when it comes to preserving the essential normativity of academic medical ethics. It does not follow from this, however, that derived, theoretical knowledge in medical ethics should be considered to enjoy some kind of epistemic superiority compared to practical knowledge arising in the lived and experienced real world practice. Rather, medical ethics research must address the different conditions for producing theoretical and practical knowledge respectively, and theoretical and practical work on ethics should be assessed according to these conditions respectively [11]. Consequently, a classification of the whole field of medical ethics should not only encompass both theoretical and empirical research but also reflect that there is a difference between arriving at academic conclusions at the desk, and arriving at practical conclusions (i.e., actions) embedded in real world practices. An adequately justified classification requires that one takes a stance towards - and provides a description of - how to perceive the relationship between academic work and impact on practice.

In our view, the relationship between real world practice and academic medical ethics can be considered as inherently dependent on each other. Real world practice can crucially influence a) the relevance of the subjects of academic approaches (by informing about what is at stake from a practical point of view), and b) the accurateness of the theoretical reflection (by providing contextualized facts). On the other hand, ethical theory and conceptual analysis can impact ethical practice by c) increasing stakeholders’ awareness of (sensitivity to) ethical claims, and d) structuring their practical conclusions according to justified norms. This means that an adequate classification of the field of medical ethics must reflect that theoretical work and empirical work on real world practices (broadly understood to encompass actual experiences of decision making around ethical dilemmas, experiences of not being treated as moral equal as well as more general concerns and challenges) are not *merely relevant* to the field of medical ethics; they are also interdependent. This puts further constraints on how the sections of our theoretical classification should be *presented*; namely that they should be presented in a way that reflects an equal importance and not a hierarchy.

## Classification: thematic perspectives versus universal features of academic work?

Finally, an adequate purposive classification requires that the categorised sections do not overlap with others in crucial respects. This means that the sections have to be structured with respect to a specific criterion *or* a set of distinct, but consistently defined, criteria that reflects a particular organisational strategy. Let us consider two ways of doing this. On the one hand, a categorisation of relevant issues in medical ethics can be presented according to substantive thematic issues. The *theme* is the specific criterion while thematic sections can be organised in different ways, e.g., according to historical development, local geographic concerns etc. Such a classification will then rely on contingent features (features that empirically could have been different). On the other hand, the classification can be structured by a set of universal (non-contingent) features of academic work, e.g., whether it is purely theoretical, applied theory on a general or a particular issue, reports on empirical experiences and perceived challenges of stakeholders in a particular setting etc.

When classifying the field of medical ethics into distinct sections, these sections might overlap, either with respect to contingent substantial themes (if we chose to structure the categorisation according to universal features of academic work), or *vice versa*. Thus, we need an argument for choosing one structuring strategy over the other.

Again, our argument is simple. There is an important reason to go with the strategy of categorising according to non-contingent features of academic work rather than to distinguish categories with respect to substantial themes. The field of medical ethics is not static; it develops dynamically in response to new compositions of contextual features that arise or new technology that is developed etc. Categorising the field of medical ethics according to already existing themes limits our capacity to recognise and find a suitable home for academic work on themes that have yet to be acknowledged or conceived.

## Summing up: justified constraints on the classification of medical ethics

We can now sum up several constraints on our theoretical classification drawn from the previous discussion. These constraints shape the classification along two different dimensions. Some constraints put *formal restrictions* on how the sections should be presented in order to expose their relative level of importance, as well as to avoid overlap between the emerging sections. Others point out *substantial areas* of the field that must be covered by a classification. We start with the formal constraints followed by the substantial areas to be presented.

First, classification of the field of medical ethics should be structured according to characteristic features of academic approaches, rather than by themes, in order to keep the categories flexibly open to new issues arising in rapidly and constantly developing conditional frames of medical ethics. This is not to say that we cannot explain and illustrate the sections with reference to particular themes. The point is that thematic descriptions should not misleadingly be presented as independent criterion for classification alongside the features of academic approaches; such a mixed presentation messes up the logic of avoiding overlap that is putatively secured by choosing one comprehensive strategy in the first place.

Second, the inherently practical structure of medical ethics requires a mix of both empirical and theoretical work. The sections in this theory-derived classification approach will have to reflect the constraint that these different approaches are formally considered to enjoy relatively equal importance in the field. A way to do this is to include approaches that can be characterised as primarily *theoretical* or *empirical* in distinct sections, and allow crossover that accommodates blending or integration of theoretical and empirical work.

Third, a substantive classification of the whole research field needs to include sections that encompass explorations of the conditions for medical ethics as a research activity itself, and this will broadly include theoretical discussions on how to establish knowledge in the field, conceptual analysis and methodological development. Proposed methodology in medical ethics would be based on such fundamental theoretical, meta-ethical work. This means that fundamental epistemological discussions and discussions about substantive methodology logically belong to different levels of justification; i.e., methodology can be derived from epistemological theories while epistemological knowledge cannot be derived from methodology. For practical reasons it may be reasonable to present all these kinds of theoretical work within the same category. For the theoretical reasons just mentioned, however, regarding logical derivation, one should be careful not to describe such an overall category in terms of the least fundamental level of justification (i.e., ‘methodology’) and indicate that the rest of the work can be seen subordinated to this term. The logic of derivation suggests it should be the other way around, whereas linguistically and conceptually a category of ‘methodology’ is easily understandable and would, at a common sense level, include epistemology as a necessary component of methodological discussion. Where logic and common sense approaches appear to clash, a compromise position may make sense; using a description broad enough to encompass any level of justification. The simplest way of doing so might be to label the category ‘epistemology and methodology’.

Fourth, as a substantial requirement there must be room in our theoretical classification for contributions of social sciences in revealing people’s experiences and opinions on medical ethics, and other disciplinary perspectives that do not deal explicitly with normative philosophical argument. This might best be described as ‘studies in empirical/interdisciplinary ethics’. The object of research may be, for example, empirical data, legal analysis, or even sociological perspectives on the field of medical ethics.

Fifth, the classification must also cover academic work that primarily addresses contextualised, empirical practices in medicine and health promotion from a theoretical point of view. This kind of work might be categorised as ‘context-dependent theoretical ethics’. This also indicates that empirical facts may be crucial components of this kind of academic work, while keeping open the question of to what extent, and in what way, the discussion is empirical/contextualized. (i.e., it allows for a range of methodological variation).

Sixth, to adequately cover the field of medical ethics research, our classification will have to include work that focuses on ethical theories or approaches that are context-independent as well. This would cover normative theories that present general ethical principles relating to medicine that in turn can be fed into context-dependent theoretical work, or ethical analysis of issues that are performed solely using theoretical approaches. This category can thereby be labelled ‘theory and analysis’.

## Theoretical classification of ‘medical ethics’

We can now sum up our theoretically derived classification of medical ethics:

- Studies in empirical/interdisciplinary medical ethics (descriptive reports on ethical issues and experiences as perceived by stakeholders)
- Context-dependent theoretical ethics (normative discussions of practical, empirical issues - informed, to a varying extent, by empirical data)
- Theory and analysis (theoretical work that justifies general ethical principles for medicine and health promotion or applies theory alone to the analysis of ethical issues)
- Epistemology and methodology in medical ethics (discussions of theoretical and empirical premises for establishing ethical knowledge and action (including conceptual analysis), and discussions of methodologies) (Table 1)

**Table 1:** Classifications of ‘medical ethics’ according to the practical, consensus-based and the theoretically driven approach, respectively

Practical, consensus-based approach to classification of ‘medical ethics’	Theoretically derived approach to classification of ‘medical ethics’
• Ethics in biomedical research • Ethics in clinical practice • Ethics in public health, medical law, and health policy • Methodology in bioethics	• Studies in empirical/interdisciplinary medical ethics (descriptive reports on ethical issues and experiences as perceived by stakeholders) • Context-dependent theoretical ethics (normative discussions of practical, empirical issues - informed, to a varying extent, by empirical data) • Theory and analysis (theoretical work that justifies general ethical principles for medicine and health promotion or applies theory alone to the analysis of ethical issues) • Epistemology and methodology in medical ethics (discussions of theoretical and empirical premises for establishing ethical knowledge and action (including conceptual analysis), and discussions of methodologies)

### Does our theoretical classification of medical ethics stand up to the test of power abuse, inadequate coverage and negative impact of theory on practice?

Our approach is a theoretically derived classification of medical ethics, originating from a desire to explore an alternative to the practical (through iterative deliberative process) classification adopted by the journal. Clearly, this interest did not arise in a vacuum of other interests, and it undoubtedly reflects particular theoretical points of view, personal experiences and opinions on what we believe medical ethics should be about that not everyone will share. If we attempted to make use of our positions to implement this classification somewhere without any open discussion, we would be guilty of power abuse. As it stands, however, we are only presenting it order to explore and demonstrate the approach-dependent challenges involved in classifying the field of medical ethics. It should also be acknowledged that what we have presented is the product of a collaboration between four different individuals, all of whom work primarily in different languages. As such, the product of our deliberation reflects the conceptual and linguistic compromises that were necessary for reaching agreement.

The classification has been developed on the condition that we can map all medical ethics research into the offered categories in an adequate way. In principle, of course, we might have overlooked relevant kinds of research that do not fit into any of our suggested categories. At this point, we are not in a position to judge the adequacy of the classification (as we have worked on it until it seemed adequate to us). From this we draw a lesson; unlike the deliberative and consensus-based approach, theoretically derived approaches to classification of medical ethics seems to require external, critical review and assessment to make sure aspect are not unknowingly neglected.

Whether this classification would have unintended adverse impact on real world practice is of course an empirical question. We have emphasised the relevance of empirical knowledge in medical ethics and the integration of empirical knowledge in theoretical approaches in the two first sections, as well as emphasising the need for meta-ethical discussions about the relationship between theory and practice in the fourth. Therefore, there should not be any undue bias towards theoretical or empirical understandings of ethics. We have, however, suggested that ultimately some theoretical understanding of ethics might come prior to being able to use empirical approaches – and this is a point that is both debatable and could be interpreted as hierarchical. Similarly, the point we make about epistemology being logically prior to methodology may be interpreted as prioritising philosophical concerns and thus to be making a political point about the relative importance of approaches within the academy. This is not intended, but could almost certainly be read into what we have said.

### Pro- and cons arguments for the theoretically derived approach

The overall advantage of classifying medical ethics using a theoretically derived approach would be that the resulting classification is consistent, and consistently justified. Moreover, the sections are deliberatively developed to *systematically* cover the field as adequately as possible. The weaknesses in this approach, however, are not insignificant. This approach results in a cumbersome overview; it may be difficult to understand (and journals may forfeit submissions for that reason alone). So this approach requires some simplification; but that comes with the potential cost of losing the adequacy of conceptual coverage.

## Conclusion

In this article we have aimed to reflect on the possibilities, challenges, and value of classifying the field of medical ethics. We have developed a classification shaped by theoretically derived constraints and conditions, which contrasts the practical, consensus-based approach to structuring the classification used by this journal. Interestingly, these different approaches reflect a fundamental tension within the field of medical ethics itself, i.e., between theoretical and practical approaches and their different ways of ensuring validity of their conclusions. The choice of approach is likely to have implications for how the field ends up being divided and presented conceptually (although the results could in principle coincide). By our assessment, both approaches have their own strengths and weaknesses respectively. As they follow their own distinct logics in how to reach a classification, it is important to perceive them as incommensurable; neither of them indicates critical assessments of the other. Overall, when it comes to determining what approach to use to classify the field of medical ethics, the path and the set of sections chosen by this journal seem best justified for practical editorial purposes. The easily understandable labelling combined with the institutionalised flexibility of editors’ judgments in finding the appropriate section for any article, mitigates against the risk of not covering the field adequately. Furthermore, the added social - and educational - value of having the editorial board collectively participating in the discussion about the classification should not be underestimated. The approach we have explored here is probably best seen as a theoretical exploration of the field. Nevertheless, this exercise has hopefully contributed to important reflection; there are different approaches to developing a classification of the field, and these may result in substantially different, although respectively valid, categories. Importantly, exploring the potential risks and weaknesses of each approach is valuable in itself – encouraging reflexivity and ensuring that people engaging in classification activity, which might seem internal and without consequence, are conscious and mindful of the potential impact of their decisions.
